# *Cystoisospora suis* – A Model of Mammalian Cystoisosporosis

**DOI:** 10.3389/fvets.2015.00068

**Published:** 2015-11-30

**Authors:** Aruna Shrestha, Ahmed Abd-Elfattah, Barbara Freudenschuss, Barbara Hinney, Nicola Palmieri, Bärbel Ruttkowski, Anja Joachim

**Affiliations:** ^1^Department of Pathobiology, Institute of Parasitology, University of Veterinary Medicine Vienna, Vienna, Austria

**Keywords:** swine, piglets, *Cystoisospora suis*, immunology, microbiome, coccidian

## Abstract

*Cystoisospora suis* is a coccidian species that typically affects suckling piglets. Infections occur by oral uptake of oocysts and are characterized by non-hemorrhagic transient diarrhea, resulting in poor weight gain. Apparently, primary immune responses to *C. suis* cannot readily be mounted by neonates, which contributes to the establishment and rapid development of the parasite, while in older pigs age-resistance prevents disease development. However, the presence of extraintestinal stages, although not unequivocally demonstrated, is suspected to enable parasite persistence together with the induction and maintenance of immune response in older pigs, which in turn may facilitate the transfer of *C. suis*-specific factors from sow to offspring. It is assumed that neonates are particularly prone to clinical disease because infections with *C. suis* interfere with the establishment of the gut microbiome. Clostridia have been especially inferred to profit from the altered intestinal environment during parasite infection. New tools, particularly in the area of genomics, might illustrate the interactions between *C. suis* and its host and pave the way for the development of new control methods not only for porcine cystoisosporosis but also for other mammalian *Cystoisospora* infections. The first reference genome for *C. suis* is under way and will be a fertile ground to discover new drugs and vaccines. At the same time, the establishment and refinement of an *in vivo* model and an *in vitro* culture system, supporting the complete life cycle of *C. suis*, will underpin the functional characterization of the parasite and shed light on its biology and control.

## Introduction

*Cystoisospora suis* (syn. *Isospora suis*), an apicomplexan parasite of swine, is the causative agent of neonatal porcine cystoisosporosis (coccidiosis). The parasite was first described in 1934 (1), but it received recognition only after the introduction of intensive, high-throughput pig breeding facilities in the mid-1970s (2–4). Suckling piglets are the most affected age group and frequently show pasty-to-watery non-hemorrhagic diarrhea and marked weight loss, while older pigs are less susceptible and excrete few or no oocysts without clinical signs upon infection. Despite high rates of morbidity, piglets exhibit high individual variability in the development of disease (5, 6), which leads to uneven weaning weights (7, 8). Infected piglets usually recover within 2 weeks post-infection (9–11). Although cystoisosporosis has a ubiquitous distribution (12–15), the diagnosis is still cumbersome because of variations in the excretion intensity (16) and short individual oocyst excretion periods (10).

Several species of the genera *Eimeria* and *Cystoisospora* can infect swine. Unlike in other livestock, where mixed infections with various *Eimeria* species are common (17–20), *C. suis* is the predominant pathogen in pigs (15, 21). Economic losses associated with coccidiosis in livestock are mainly due to impaired performance, retarded growth, mortality, and cost of treatment. Moreover, cystoisosporosis is thought to predispose the piglet to infection with secondary bacterial and viral pathogens, which subsequently increase morbidity, mortality, and managerial costs (22). There are no vaccines available so far, and toltrazuril is the only licensed drug for metaphylaxis that can effectively suppress oocyst excretion and improve piglet health both under experimental conditions (8, 23) and in the field (24). However, rapid emergence of resistance against all introduced anticoccidials in chicken *Eimeria* (25) is also of concern regarding porcine cystoisosporosis, and there is an urgent need to develop new and sustainable intervention strategies against *C. suis* for combating neonatal porcine cystoisosporosis in the future.

An experimental model mimicking the field situation (10) in conventional piglets gave deeper insight into neonatal porcine cystoisosporosis. This was further strengthened by the establishment of an *in vitro* culture system supporting the entire lifecycle of *C. suis* in intestinal porcine epithelial cells (26). Moreover, gnotobiotic piglets are available as infection models for specific applications (3, 21). Taken together, *C. suis* may serve as a representative infection model for comparative research on mammalian cystoisosporosis.

## *Cystoisospora* – What Do We Really Know about the Life Cycle?

**Figure 1:**
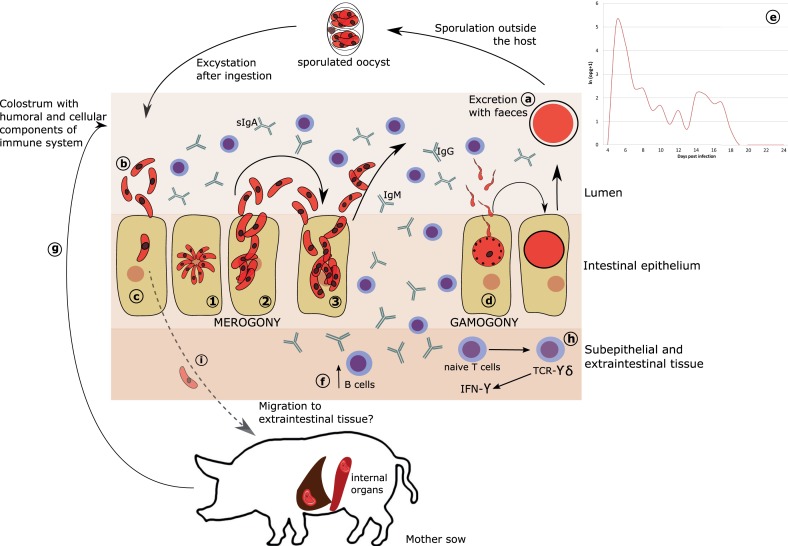
**Proposed model of *C. suis* development and immunity**. **(A)** Oocysts are excreted with feces and undergo sporulation in the environment. **(B)** Sporulated oocysts excyst upon ingested by host to release sporozoites. **(C)** Sporozoites invade intestinal epithelium and develop to become merozoites (1). In contrast to *Eimeria*, merogony in *C. suis* is not synchronized but rather stages are defined as types (2–3). It is currently not known which type could act as extraintestinal resting stage. **(D)** Merogony is followed by gamogony resulting in fusion of macro- and micro-gametes to form a zygote and subsequently an oocyst. **(E)** The desynchronization of the merogonic development may also be responsible for the characteristic oocyst excretion occurring in two (or more) peaks, when some of the merozoites may undergo rapid development to gamogonic stages, while others enter into a development lag phase to undergo the sexual maturation for the next peak. **(F)** In response to infection, naïve B cells proliferate and produce IgA, IgG, and IgM. **(G)** Intake of colostrum and milk, rich in antibodies and cellular components could partially confer passive humoral immunity against *C. suis* from sow to piglets. **(H)** Following infection, TCR-γδ T cells show an almost 30-fold increase in the epithelium and are assumed to be the major producers of IFN-γ, which could support the termination of primary infection in pigs harboring sufficient numbers of these cells in the gut, which is age dependent. **(I)** The existence of extraintestinal stages of *C. suis* in liver and spleen of adult pig has been proposed, but viable stages in these tissues have not been demonstrated yet.

Like other *Cystoisospora* species, *C. suis* entirely develops in one host (26, 27) (Figure 1). Directly after ingestion, sporulated oocysts undergo excystation and sporozoites invade the small intestine epithelium (12, 28) to reproduce within a parasitophorus vacuole (29, 30). Asexual reproduction (merogony) peaks at day 4 and 5 post-infection. Unlike *Eimeria*, merogonic stages are not assigned to generations but to types defined by the number of nuclei, shape, size, and time of appearance (26, 27, 31). From day 5, mature sexual stages can be identified (3, 31). After fusion to form a zygote, the unsporulated oocyst is excreted with the feces and undergoes sporogony outside the host (27, 28, 32).

Various environmental conditions influence the sporulation time. Lindsay et al. (33) found that the most rapid sporulation takes place between 30 and 37°C, which is well supported by the conditions prevailing in a modern farrowing unit. Rapid multiplication of sporozoites and merozoites inside the intestinal epithelium leads to massive histological alterations including atrophy, necrosis, and fusion of villi, hyperplasia of crypts, and desquamation of epithelial cells (12, 29, 34, 35). These changes persist for a considerable time after parasite development (8), which may contribute to the reduction in body weight gain due to lasting impairment of nutrient absorption.

*Cystoisospora suis* completes its life cycle within 5–6 days (36). Clinical signs can be seen as early as 3 days post-infection (dpi), shedding of oocysts typically starts on fifth dpi (6, 10, 21, 28, 31, 35). However, these periods may differ, probably due to the age and health condition of the piglets and the virulence of the parasite strain (3, 10, 35). Oocyst excretion and symptoms show typical peaks at 5th–9th and 11th–14th dpi (21, 28, 30), which might be due to extraintestinal stages re-entering the intestines (3).

It has been shown for several *Cystoisospora* species (*C. felis*, *C. rivolta*, *C. canis*, and *C*. *ohioensis*) that sporozoites enter extraintestinal tissues, most often mesenteric lymph nodes but also liver, spleen, other lymph nodes or skeletal muscle, and form monozoic cysts. These extraintestinal stages have been found in definitive as well as in paratenic hosts (3, 27, 37). Also, *C. belli* extraintestinal cysts were described in humans (38). Paratenic hosts do not show clinical signs but act as carriers, since parasites can survive for at least 2 years within their tissues (32).

However, no study could so far unequivocally demonstrate the existence of *C. suis* extraintestinal stages in infected piglets or in potential paratenic hosts. Previous studies (31, 37, 39) could not provide evidence of extraintestinal stages in tissues of experimentally infected piglets or mice. Still, gnotobiotic piglets shed oocysts after intraperitoneal inoculation of liver, spleen, and lymph node homogenates from experimentally infected piglets (3).

In a preliminary study, *C. suis*-specific PCR of tissues from experimentally infected piglets revealed the presence of parasite DNA in several organs. In spleen and mesenteric lymph nodes, it could first be detected on the second dpi. In kidney tissues, it was detected on the second and in kidney and liver tissue from the fifth to the ninth dpi. In jejunal mucosa, it was found from the 1st dpi until the end of the study on 13th dpi (40). Although detection of DNA does not prove the presence of viable, infectious parasitic cells, these results indicate trafficking of *C. suis* to extraintestinal tissue, either by active migration or after phagocytosis, e.g., by macrophages, and still warrant further studies.

## Immunity and Age Resistance Against *Cystoisospora* – What is What?

Many aspects, such as age, maturation of the gut immune system, as well as the immune status of the infected piglet, influence resistance to *C. suis*. Stuart et al. (41) showed that piglets infected during the first 3 days of their life develop severe clinical signs compared to 2-week-old infected piglets. Also, when piglets were infected at 3rd vs. 9th day of their life (or on both days) with high doses of *C. suis*, the clinical signs and oocyst output were most notable in early infection, while piglets infected on the 3rd and on 9th day of life and those infected for the first time on the day 9th of life did not significantly differ (42). Therefore, the authors concluded that age resistance (based on the maturation of the innate immune system) plays a more important role than acquired immunity. Age resistance seems to be a general feature in coccidiosis and is probably due to an increase of T cells and IFN-γ production in the spleen of mice with increasing age that became resistant to primo infections with *Cryptosporidium parvum* (43). However, in some mammalian species, susceptibility increases with the age of the animal before it decreases again (44, 45), which may also be related to changing immune responses in older animals (46).

Maturation of the porcine immune system can also influence the clinical outcome. Piglets are born with a premature immune system, which only starts to develop during the first few weeks of age. Neonatal piglets do not have well-developed Peyer’s patches; CD2^+^CD4^−^ and CD8^−^ T cells make most of the intraepithelial lymphocytes and CD8^+^ cells are not present until the seventh week of life. Also, T cells within the lamina propria of the small intestine and interfollicular areas of Peyer’s patches were found to be fewer compared to older pigs. Likewise, the small intestinal mucosa of new-born piglets is characterized by the absence of lymphoid cells with the exception of a few antigen presenting cells and T cells (47), which may explain the severity of the disease in young piglets due to the inability to adequately respond to the parasite. In older piglets, by contrast, Worliczek et al. (16) detected changes in the T-cell populations of infected piglets, which displayed decreased cell numbers in blood, spleen, and mesenteric lymph nodes and increased T-cell numbers in the epithelium and the lamina propria of the jejunum of *C. suis* infected piglets, indicating a specific immune response to infection. The most prominent subpopulation in the gut epithelium was T-cell receptor-γδ (TcR-γδ) cells, which are engaged in the primary immune response to pathogens (16, 34). TcR-γδ T-cells were also found to be involved in the immune response against other coccidian parasites, e.g., *Eimeria vermiformis* of mice (48).

For other coccidian parasites, humoral immune response seems to have a minor role in the protection mechanism. Schito et al. (49) suggested that primary infection with different *Eimeria* spp. is controlled by innate immune response. Stimulation of humoral immunity by *Eimeria* is known but its effectiveness in controlling the infection is still unclear (50). Immune sera from *E. tenella*-infected chicken and *E. falciformis* in mice enhanced the phagocytic activity of macrophages (51, 52). In spite of the fact that piglets are born with an immature immune system (47), cellular immune responses might be involved in the development of immunity against coccidian parasites including *C. suis* (16, 53–55). The role of passive immune response and the transmission of immune components from infected sows to piglets had been neglected by many authors (41, 56, 57). However, earlier works have shown that colostral antibodies may participate in resistance against natural infections with *C. suis* (58, 59). Recently, Schwarz et al. (60) demonstrated that naturally acquired *C. suis*-specific antibodies (IgA, IgM, and IgG) were transferred from sows to their piglets via colostrum, which in turn provided partial protection against the outcome of experimental infection (clinical disease and oocyst shedding) in the presence of high IgA titers in colostrum as well as milk and serum of superinfected sows. It is currently unclear whether the detected immunoglobulins have a protective function by themselves or are merely markers for protection conveyed by other, not yet explored, mechanisms.

## Can Comparative Genomics Help to Unravel Biology and Support New Intervention Strategies?

While the genomes of many coccidian species are available in the ToxoDB database (61), *C. suis* is still lacking a reference genome. Moreover, the number of chromosomes is also unknown. To date, only few ribosomal and mitochondrial sequences of *Cystoisospora* species were generated for phylogenetic studies, which established that the genus *Cystoisospora* constitutes a monophyletic clade with the Sarcocystidae, and it is closely related to *Toxoplasma* and *Neospora* (62–64). These studies also confirmed the hypothesis that heteroxeny is an evolutionary derived character in *Cystoisospora* (62).

Current Next Generation Sequencing (NGS) technologies allow assembling of new genomes in a rapid and inexpensive way. First estimates based on NGS data showed that the genome of *C. suis* is about 84 Mb and contains more than 8000 genes (65). These numbers are comparable to other coccidian species; however, comparative genomics analyses revealed that only about 60% of the *C. suis* genes have orthologs in *T. gondii* (65), implying a greater divergence than expected between these two species. Thus, to generate a comprehensive gene catalog of *C. suis* it will be crucial to integrate gene predictions with RNA-Seq data from different developmental stages. This will also allow for identification of genes involved in life stage transitions, as similarly performed in *E. tenella* (66). Finally, RNA-Seq can elucidate the molecular changes of *C. suis* and pig during infection, as exemplified in experiments in *N. caninum* (67) and *T. gondii* (68).

Intervention strategies can also be aided by genomics. Currently, the drug toltrazuril is the only treatment available against *C. suis*; however, resistance has already emerged in *Eimeria* (69), implying the necessity to find new effective drugs. The availability of the gene catalog of *C. suis* will be a starting point to detect drug targets, based on the functional annotation of protein-coding genes. Typically, annotation of gene function can be inferred on the basis of orthologous proteins, using tools such as Blast2GO (70). Afterwards, screening for drug targets can be performed on the basis of the functions of candidates identified as drug targets in other coccidia. These include protein kinases (71–73), apicoplast proteins (74), enzymes involved in fatty acid biosynthesis (75) and shikimate metabolism (76), mitochondrial proteins (77), and others, reviewed in Ref. (78). Another approach to identify drug targets involves comparing the metabolic pathways of parasite and host (79), for example, selecting pathways that are present in *C. suis* but absent in pig.

**Figure 2:**
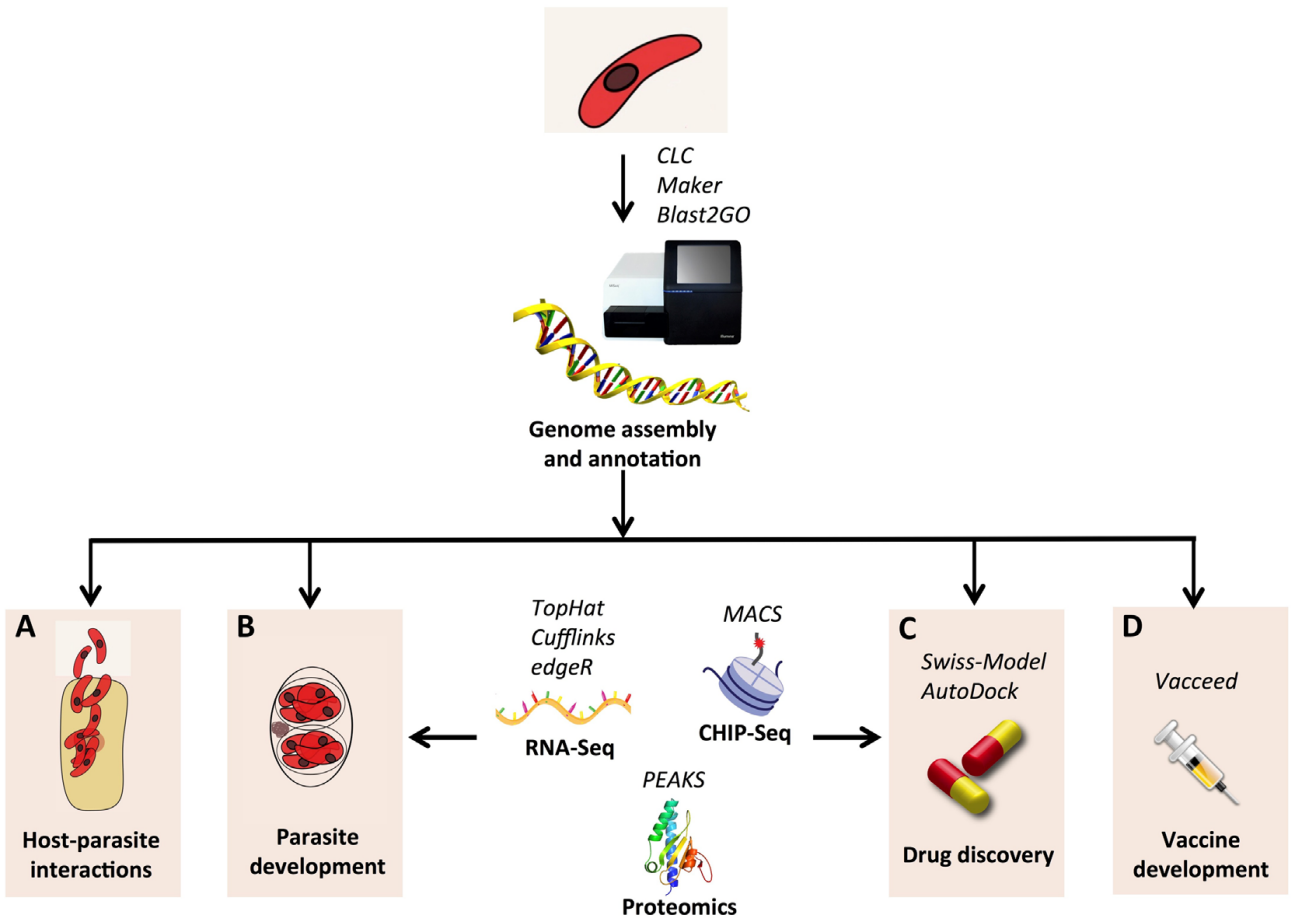
**Schematic view of the *in silico* analysis of genomic data for *C. suis***. The genome sequence can be assembled with next generation sequencing using a combination of short and long reads libraries. Tools such as CLC (CLC Bio-Qiagen, Aarhus, Denmark), Maker (88), and Blast2GO (70) can be applied for *de novo* assembly, annotation, and functional annotation, respectively. Other NGS technologies, such as RNA-Seq (89) and CHIP-Seq (90), together with proteomics (91), can be used to unravel the biology of the parasite and to discover new drugs and vaccines. Changes involved in host–parasite interaction **(A)** and developmental switches **(B)** can be identified both at the genetic and epigenetic level by RNA-Seq and CHIP-Seq, respectively: transcripts are reconstructed using the programs TopHat (92) and Cufflinks (93); differentially expressed genes are detected by edgeR (94); CHIP-Seq data are processed with the MACS software (95). **(C)** 3D structure of drug candidates can be reconstructed by homology using Swiss-Model (96); screening of virtual libraries of compounds can be performed with AutoDock (97). **(D)** Vaccine candidates can be identified using Vacceed (86) and validated by proteomics approaches, such as mass spectrometry, with the aid of the software PEAKS (Bioinformatics Solutions Inc., Waterloo, ON, Canada).

An alternative control route might be vaccination; however, there is at present no vaccine available against *C. suis*. Although early attempts using the merozoite attachment protein SAP induced a 96–99% reduction in merozoites (80), the resulting vaccine patent was withdrawn. In this regard, genomics can also contribute to vaccine discovery: using the reverse vaccinology paradigm (81), it is possible to screen the genome for vaccine candidates by identifying proteins with immunogenic features. This approach has been successfully applied in various bacterial species (82). However, the inherent complexity of eukaryotic pathogens has hindered the application of this strategy in such organisms. Recently, the feasibility of reverse vaccinology has been reviewed in the coccidian parasite *N. caninum* (83). In parallel, bioinformatics tools and pipelines have finally emerged to address the specific issue of detecting vaccine candidates in eukaryotic pathogens (84–87). An overview of the *in silico* analysis of the *C. suis* genomics data is depicted in Figure 2.

## Interactions of *C. suis* with the Gut Microbiota

The gut ecosystem is maintained by close cross-talk between host, intestinal microbiota, and parasites (98), and ultimately this has implications on host health and diseases (99). Excretory and secretory products of intestinal parasites may continuously disrupt the balance between the gut microbiota and the body (100), whereas on the other hand, metabolic products of the microbiota may also interfere with the establishment and survival of parasites, subsequently changing the outcome of parasitic infection (100).

The digestive tract of piglets is sterile at birth and becomes rapidly colonized with microorganisms from the surrounding environment (101, 102). Strict anaerobes predominate in the normal flora and this microbial composition and diversity underpins the health status of the pigs (103, 104), especially during the suckling and post-weaning period. Symbiotic interactions between host and gut microbiota mainly occur along the intestinal mucosa (105). Since *C. suis* is mainly localized in the intestinal mucosa, more precisely in the epithelial cells of the villi and, in heavier infections, also the crypts (12), it is prudent to assume that it may strongly interact with the gut microbiota of the host. It is well documented that coccidiosis in chickens highly influences the diversity of gut microbiota (106–108). Damage of intestinal epithelium during intracellular multiplication of *Eimeria* enhances mucus secretion from goblet cells together with leakage of glycoproteins and mannose residues, which favors growth and adherence of pathogenic bacteria-like *Clostridium perfringens* (109, 110) and *Salmonella typhimurium* in germ-free chickens (111). More recently, Kirino et al. (112) reported significantly higher *Eimeria* OPG count in fecal samples of Japanese beef cattle suffering from hemorrhagic enteritis compared to the control animals. Based on microbiological examination, the authors also found that the mean fecal coliform count was also significantly higher in the cattle harboring both *Eimeria zuernii* and *Cl. perfringens*.

Cystoisosporosis is characterized by high morbidity and low mortality within a litter. Increased mortality, however, may be related to coinfection and/or secondary bacterial infection (103, 113). Entry of pathogenic microorganism following disruption of mucosal barrier as a result of multiplication of *C. suis* has also been demonstrated in pigs (114). Results obtained by Mengel et al. (103) highlighted a correlation between clostridial infection and clinical cystoisosporosis, which further confirms the hypothesis that *C. suis* creates a suitable environment for extensive development of *Cl. perfringens*, as severe clinical signs and mortality occurred only in pigs that harbored both pathogens.

Klaus (115) examined the fecal flora of piglets from three groups, one infected with *C. suis* on the first day of life, one infected with *C. suis* and treated with toltrazuril 2 days later, and one uninfected group. It was evident that the fecal flora of young piglets undergoes significant changes during the first weeks of life, with an initial high excretion of *E. coli* and other enterobacteriaceae, followed by an increase of lactobacilli, which appeared to stabilize the intestinal environment. Irrespective of treatment groups, high numbers of enterococci were excreted during the period of parasitic invasion. The average excretion of *Cl. perfringens* was highest in the infected untreated group and lowest in the uninfected animals, indicating that infection with *C. suis* seems to alter the succession of bacterial colonization and that this effect can be partially reversed by toltrazuril treatment (115). These results are in accordance with a study conducted by Alnassan et al. (116), where prophylactic medication of chickens with toltrazuril before infection caused less severe coccidial and subsequent necrotic enteric lesions in treated individuals. Further research on the development of the gut microbiota during the first weeks of life is needed to understand the role of bacterial colonization in the pathogenesis of coccidiosis in young animals including piglets.

Moreover, as pigs may serve as an animal model for many human pathologies (117), interactions between *C. suis*, the gut microbiota, and the intestinal immune system in piglets may also help to understand the pathogenesis of other neonatal diarrheal diseases in mammals.

## Conclusion

Although current knowledge on the immunity and host–­pathogen interactions of neonatal porcine cystoisosporosis is still fragmentary, recent findings indicate that sustainable control must focus on immunity-based methods and new drug targets, taking into consideration the interaction of the parasite with the gut microbiome. As the immature immune system in new-born piglets seems to be incapable of controlling the parasite, the role of maternal immunity should be reconsidered. The presence of a single available compound against cystoisosporosis calls for urgent development of new drugs and vaccines as sustainable control methods against *C. suis*. We prospect that genomics and transcriptomics analyses will certainly play a major role in finding new drug targets and vaccines. Moreover, since *C. suis* significantly disturbs the composition of the microbial gut community, intervention strategies must focus on a more holistic approach to piglet health. We anticipate that a deeper understanding of the biology *C. suis* will favor the flourishing of studies in other mammalian hosts, where coccidiosis is often enigmatic and frequently neglected. Since new tools are available to carry out research on porcine cystoisosporosis, we propose that *C. suis* can serve as a model for cystoisosporosis in other mammals.

## Author Contributions

AS drafted the introduction and compiled the manuscript; AA-E compiled the chapter on immunology and age resistance, BF drafted the chapter about the life cycle, NP drafted the chapter about the genomics analyses, AJ, BR, and BH devised the outline, added unpublished data, and revised and edited the MS together with all other authors.

## Conflict of Interest Statement

The authors declare that the research was conducted in the absence of any commercial or financial relationships that could be construed as a potential conflict of interest.
